# The history of necrotising otitis externa: from clinical observation to conceptual recognition

**DOI:** 10.1017/S0022215126105210

**Published:** 2026-08

**Authors:** Vasiliki Bisbinas, Emma Stapleton

**Affiliations:** 1 Department of Otolaryngology, Manchester Royal Infirmary, UK; 2 Manchester Academic Health Sciences Centre, The University of Manchester Faculty of Biology Medicine and Health, UK

**Keywords:** diabetes mellitus, immunosuppression, osteomyelitis, otitis externa

## Abstract

**Objectives:**

Necrotising otitis externa is a severe, potentially life-threatening infection of the external auditory canal and skull base, first formally described by Chandler in 1968. However, historical literature suggests that its clinical features were recognised long before this, without conceptual unification.

**Methods:**

A narrative historical review was undertaken using primary and secondary sources, including early otological texts, case reports and modern literature.

**Results:**

Descriptions consistent with necrotising otitis externa appear as early as the nineteenth century, with recurrent observations of severe otitis externa, temporal bone involvement and systemic vulnerability. These remained fragmented across diagnostic categories until the mid-twentieth century. Chandler’s work represents a transition from clinical observation to conceptual recognition, providing a unifying framework that enabled consistent diagnosis, comparison and subsequent study of the condition.

**Conclusion:**

The evolution of necrotising otitis externa reflects a process of progressive recognition, offering insight into ongoing challenges in defining and studying the disease in contemporary clinical practice.

## Introduction

Necrotising otitis externa (NOE) is a severe and potentially life-threatening infection of the external auditory canal, characterised by progressive spread to the temporal bone and skull base, with associated risks of cranial neuropathy, intracranial extension and death.^1^^,^^2^ First comprehensively described by James R Chandler in 1968 as “malignant external otitis”,^3^ the condition is now more commonly referred to as necrotising otitis externa, reflecting its invasive and destructive pathophysiology rather than any neoplastic implication. NOE predominantly affects vulnerable patient groups, particularly those with advanced age, diabetes mellitus, immunosuppression or compromise of the external auditory canal epithelium.^2^^,^^4^)

The clinical burden of NOE is considerable. Patients frequently experience severe pain, persistent infection and prolonged treatment courses, often requiring repeated imaging, long-term antimicrobial therapy and inpatient care.^5^^,^^6^ The condition carries a significant risk of complications and has a profound impact on quality of life.^7^ From a health systems perspective, NOE represents a growing challenge, with increasing incidence observed in the United Kingdom and elsewhere, likely reflecting demographic changes including an ageing population and rising prevalence of diabetes mellitus.^8^^,^^9^ Despite this, heterogeneity in diagnostic criteria, terminology and outcome reporting has limited the comparability of studies and the development of robust evidence to guide management.^4^^,^^10^

Although NOE is widely attributed to Chandler’s description in 1968, this attribution reflects a broader issue in medical history: the distinction between the clinical existence of a disease and its formal conceptual definition. Contemporary literature continues to highlight inconsistencies in diagnostic criteria, terminology and outcome measures, underscoring that NOE remains incompletely defined even today.^4^^,^^10^

The analysis of historical literature provides an important framework through which to explore this problem. Examining early descriptions of disease allows identification of clinical patterns that may predate formal classification and offers insight into how diagnostic entities emerge over time. In otology and wider medicine, retrospective analysis of historical texts has been shown to refine understanding of disease evolution, challenge assumptions regarding priority of discovery and illuminate the influence of scientific and cultural context on medical knowledge.^11^^–^^13^ Such an approach is particularly relevant in conditions like NOE, where definitional ambiguity persists despite advances in modern practice.

This paper argues that the disease process now recognised as NOE existed in clinical practice long before its formal definition. However, it remained conceptually fragmented across diagnostic categories. The history of NOE, therefore, is not one of discovery, but of progressive recognition.

## Materials and methods

This study was conducted as a narrative historical review of the medical literature relating to necrotising otitis externa and its antecedent descriptions. The aim was not to perform a systematic review, but to identify and analyse key primary and secondary sources that contribute to the understanding of the historical development of this disease entity.

Primary sources included original case reports and early clinical descriptions of external ear disease, temporal bone osteomyelitis and related conditions published from the early nineteenth century onwards. Secondary sources comprised otological textbooks, historical reviews and modern analyses of necrotising otitis externa.

Relevant material was identified through a combination of archival texts, institutional and personal library collections and indexed electronic databases including PubMed. Reference lists of retrieved articles were reviewed to identify additional pertinent sources. Where available, original publications were consulted directly; in cases where primary texts were not accessible, secondary citations were used with appropriate caution.

Given the absence of a consistent historical definition of necrotising otitis externa, inclusion of sources was based on the presence of clinical features consistent with current understanding of the disease, including invasive otitis externa, progression to osteomyelitis of the temporal bone or skull base and association with systemic vulnerability such as diabetes mellitus or immunosuppression.

Sources were analysed qualitatively, with emphasis placed on identifying recurring clinical patterns, terminology and conceptual frameworks. Particular attention was given to the evolution of disease classification and the point at which disparate observations were synthesised into a recognisable clinical entity.

## Results

### Early descriptions: recognition of disease without conceptual unity

Descriptions consistent with NOE can be identified in the medical literature as early as the nineteenth century. Toulmouche (1838) described cases of otorrhoea associated with progressive temporal bone involvement, suggestive of invasive infection extending beyond the external auditory canal.^14^ Although the original text is not readily accessible, subsequent citations indicate that severe complications of ear disease were recognised at this time.

Throughout the nineteenth century, authors documented severe inflammatory and suppurative diseases of the external ear, often associated with tissue destruction and functional impairment. Hooper (1809) described progressive inflammation leading to ulceration and loss of hearing,^15^ while von Tröltsch (1874) detailed the extension of ear disease to the temporal bone and its potentially fatal consequences.^16^ These early accounts demonstrate clear recognition of aggressive disease processes involving the external ear.

By the early twentieth century, further descriptions emerged of complications extending beyond the ear canal. Grayson (1907) reported intracranial involvement associated with chronic ear disease,^17^ and Laurens (1919) described “diffuse external otitis” as a severe and generalised inflammatory condition of the ear canal.^18^ These observations suggest that clinicians were encountering invasive infections consistent with modern descriptions of NOE.

However, these accounts were not unified into a single diagnostic entity. Instead, they were interpreted within broader categories of otitis externa, chronic suppuration or complications of ear disease. The absence of a distinct conceptual framework meant that these cases were recognised clinically, but not defined as a specific disease.

### Mid-twentieth century: emergence of defining clinical features

By the mid-twentieth century, several features now considered characteristic of NOE had been repeatedly observed. These included association with diabetes mellitus, infection with *Pseudomonas aeruginosa* and progression from otitis externa to osteomyelitis of the temporal bone.

Meltzer and Kelemen (1959) described a case of “pyocyaneous osteomyelitis” involving the temporal bone, mandible and zygoma in a patient with uncontrolled diabetes.^19^ The disease followed a progressive course from persistent otitis externa to extensive osteomyelitis, ultimately resulting in death. The authors identified key features including the diabetic background, the presence of *Pseudomonas* and pathological bone involvement, closely aligning with modern understanding of NOE.

Other contemporaneous descriptions further support this pattern. Simpson (1957) described an infiltrative form of diffuse infective otitis externa involving deeper structures,^20^ while Collins (1964) noted that acute diffuse otitis externa could extend into the bony meatus.^21^ Earlier authors had also highlighted the role of trauma, moisture and infection in predisposing to severe disease.^18^^,^^20^

Despite this accumulation of clinical insight, terminology remained inconsistent and fragmented. Conditions were described using a variety of terms, including “diffuse otitis externa,” “deep infection” and osteomyelitis, without recognition of a unifying disease process. This reflects a stage in which the clinical syndrome was increasingly recognisable, but had not yet been conceptualised as a discrete entity.

### Chandler and the consolidation of a clinical entity

A critical shift occurred with Chandler’s description of “malignant external otitis” in 1968.^3^ Chandler identified a consistent clinical pattern characterised by elderly or diabetic patients presenting with otitis externa that progressed to invasive infection of the temporal bone and skull base.

Importantly, the features described by Chandler had been observed previously. His contribution lay in recognising their relationship and defining them as a single disease entity. By synthesising disparate observations into a coherent clinical framework, Chandler established a diagnostic concept that could be consistently recognised, studied and managed.

This represents a transition from clinical observation to conceptual recognition, rather than the initial identification of a novel disease.

### Evolution of terminology and ongoing conceptual variability

Following Chandler’s work, terminology evolved, with increasing use of the term necrotising otitis externa.^22^ This shift was in part a response to concerns that the term “malignant” was potentially misleading, implying neoplastic disease rather than invasive infection.

However, terminology has remained variable. Historical and contemporary literature demonstrate continued use of both malignant and necrotising otitis externa, and variation persists across international practice.^1^^,^^23^ Early references to “necrotic” otitis externa also appear in non-English literature from the 1970s, suggesting parallel developments in terminology.^23^

This variability reflects the ongoing evolution of the conceptual framework of NOE. Despite increasing recognition, there remains no universally consistent definition, and the boundaries of the disease continue to be refined in current literature.^4^

### Reinterpretation of historical literature in light of modern understanding

When viewed through the lens of contemporary knowledge, many earlier descriptions can be reinterpreted as representing NOE. References to severe otitis externa with deep tissue involvement, osteomyelitis and intracranial complications likely describe the same underlying disease process.

However, these cases were historically classified according to the prevailing frameworks of the time, which emphasised anatomical location or isolated pathological features. As a result, the disease was fragmented across multiple diagnostic categories.

This retrospective reinterpretation highlights a key finding of the present analysis: the clinical syndrome now recognised as NOE was repeatedly encountered and described, but lacked the conceptual framework required for its definition as a distinct entity.

These findings are summarised in Table 1, which illustrates the evolution of the conceptualisation of necrotising otitis externa from early clinical observation to ongoing definitional refinement.

**Table 1.** Evolution of the conceptualisation of necrotising otitis externa

Period	Conceptual understanding	Key features described	Interpretation
Nineteenth century	Severe external ear disease recognised	Otorrhoea, tissue destruction, extension beyond ear canal^14^^–^^16^	Clinical features present, no unifying disease concept
Early twentieth century	Recognition of complications	Intracranial extension, temporal bone involvement^17^^,^^18^	Viewed as complications of ear disease rather than a distinct entity
Mid-twentieth century	Emergence of defining features	Diabetes association, *Pseudomonas* infection, osteomyelitis^19^^–^^21^	Increasing pattern recognition without conceptual consolidation
1968	Conceptual consolidation	“Malignant external otitis” defined^3^	Unification of disparate observations into a clinical entity
Late twentieth century	Terminological evolution	Shift from “malignant” to “necrotising” otitis externa^22^^,^^23^	Movement towards pathophysiological description
Modern era	Ongoing definitional refinement	Evolving consensus definitions and outcome sets^4^^,^^10^	Recognition that disease conceptualisation remains incomplete and evolving

## Discussion

The findings of this study support a central conclusion: NOE did not emerge suddenly as a newly observed disease in the mid-twentieth century, but rather represents the culmination of a prolonged process of clinical recognition. Historical sources demonstrate that the key features of NOE, including invasive infection, osteomyelitis of the temporal bone and association with systemic vulnerability, were repeatedly observed over more than a century prior to their formal definition. What was absent was not clinical awareness, but conceptual unification.

This distinction between observation and recognition is critical. In the absence of a coherent diagnostic framework, early descriptions of severe external ear infection were dispersed across multiple categories, including diffuse otitis externa, chronic suppuration and osteomyelitis. These classifications reflected the prevailing structure of medical knowledge, which tended to prioritise anatomical localisation over disease trajectory. As a result, the underlying syndrome remained obscured despite being clinically apparent.

Chandler’s contribution can therefore be understood not as the discovery of a new disease, but as a point of conceptual consolidation. By identifying a consistent pattern of clinical features and defining it as a distinct entity, he transformed a series of fragmented observations into a recognisable diagnosis. This process whereby an existing clinical phenomenon is retrospectively unified and named is well recognised in the history of medicine and underpins the evolution of many disease concepts.

Importantly, the historical trajectory of NOE has clear parallels in contemporary practice. Despite advances in imaging, microbiology and multidisciplinary care, there remains no universally accepted definition of NOE, and variability persists in diagnostic criteria, terminology and outcome reporting.^4^^,^^10^ This suggests that the process of conceptual refinement is ongoing. The persistence of terms such as “malignant” and “necrotising” otitis externa, often used interchangeably, reflects continuing uncertainty regarding the boundaries and essential features of the disease.

The reinterpretation of historical literature through a modern lens also highlights the limitations of retrospective diagnosis. While many early descriptions are strongly suggestive of NOE, they cannot be definitively classified as such. This is not a weakness of the historical record, but rather a reminder that disease entities are shaped by the frameworks within which they are understood. The identification of NOE in earlier texts, therefore, is necessarily interpretative, reinforcing the importance of caution in attributing priority of discovery.

From a broader perspective, this study illustrates the value of historical analysis in informing contemporary clinical understanding. By examining how disease concepts evolve, it becomes possible to identify the factors that facilitate or hinder their recognition. In the case of NOE, delayed conceptualisation appears to reflect both the heterogeneity of clinical presentation and the absence of unifying diagnostic criteria. These same challenges continue to influence modern research and clinical practice.

This has practical implications. Efforts to standardise definitions, such as recent consensus work on diagnostic criteria and core outcome sets, represent a continuation of the process initiated by Chandler. The history of NOE suggests that such efforts are essential for advancing understanding, improving comparability between studies and ultimately enhancing patient care.

The history of necrotising otitis externa, therefore, serves as a reminder that the boundaries of disease are neither fixed nor inherent, but constructed through clinical observation, interpretation and consensus. Recognising this may help to guide future efforts to refine its definition and management.

- Necrotising otitis externa (NOE) existed in clinical practice and published literature long before its formal definition
- Early literature documents key features of the disease, including invasive infection and temporal bone involvement, without conceptual unification
- Chandler’s 1968 description represents consolidation of an existing clinical syndrome rather than its discovery
- Terminology and diagnostic criteria for NOE remain variable in contemporary practice
- Historical analysis highlights the importance of conceptual clarity in disease definition and research

## Conclusion

NOE is a disease whose clinical features have been recognised for nearly two centuries, yet its definition as a distinct clinical entity is comparatively recent. The historical record demonstrates that invasive external ear infection, temporal bone involvement and association with systemic vulnerability were repeatedly observed long before they were unified under a single diagnostic framework.

The contribution of Chandler, therefore, was not the discovery of a new disease, but the recognition and consolidation of an existing clinical syndrome. This distinction highlights the importance of conceptual clarity in the evolution of medical knowledge.

Despite advances in contemporary practice, variability in terminology and diagnostic criteria persists, suggesting that the process of defining NOE remains incomplete. Understanding its history as a disease that preceded its definition provides a useful perspective for ongoing efforts to refine its classification, improve comparability in research and optimise patient care.
